# Characteristics of successful integrated family planning and maternal and child health services: Findings from a mixed-method, descriptive evaluation

**DOI:** 10.12688/f1000research.17208.2

**Published:** 2020-01-13

**Authors:** Anne Pfitzer, Christina Maly, Hannah Tappis, Mark Kabue, Devon Mackenzie, Sadie Healy, Vineet Srivastava, Gathari Ndirangu

**Affiliations:** 1 Maternal and Child Survival Program, Jhpiego, Washington, DC, 20036, USA; 2 Jhpiego, Baltimore, MD, 21231, USA; 3 Molloy Consultants, Cincinnati, OH, 45208, USA; 4 Jhpiego, New Delhi, 110020, India; 5 Jhpiego, Lusaka, Zambia

**Keywords:** family planning, postpartum, health services, integration, maternal and child health

## Abstract

**Background: **Most postpartum women in low- and middle-income countries want to delay or avoid future pregnancies but are not using modern contraception. One promising strategy for increasing the use of postpartum family planning (PPFP) is integration with maternal, newborn and child health (MNCH) services. However, there is limited evidence on effective service integration strategies. We examine facilitators of and barriers to effective PPFP integration in MNCH services in Kenya and India.

**Methods: **We conducted a cross-sectional, mixed-method study in two counties in Kenya and two states in India. Data collection included surveying 215 MNCH clients and surveying or interviewing 82 health care providers and managers in 15 health facilities across the four sites. We analyzed data from each country separately. First, we analyzed quantitative data to assess the extent to which PPFP was integrated within MNCH services at each facility. Then we analyzed qualitative data and synthesized findings from both data sources to identify characteristics of well and poorly integrated facilities.

**Results: **PPFP integration success varied by service delivery area, health facility, and country. Issues influencing the extent of integration included availability of physical space for PPFP services, health workforce composition and capacity, family planning commodities availability, duration and nature of support provided.

**Conclusions: **Although integration level varied between health facilities, factors enabling and hindering PPFP integration were similar in India and Kenya. Better measures are needed to verify whether services are integrated as prescribed by national policies.

## Introduction

In low- and middle-income countries, nearly one-fifth of birth intervals are less than 24 months, increasing health risks for both mothers and children ^1– 3^. A large proportion of these births likely results from unintended pregnancies among women in the first two years postpartum ^4^. Worldwide, most women want to avoid or delay another pregnancy for a couple of years after having a baby, but many are not using contraception ^4^.

The promotion of postpartum family planning (PPFP) is a strategy for addressing unmet needs for contraception by helping women to start a chosen contraceptive method soon after birth and either continuing with this method or choosing another to use for at least two years. Increasingly, the integration of PPFP from pregnancy to the extended postpartum period (of two years following childbirth) is seen as a promising strategy for increasing the availability and utilization of PPFP services ^5– 7^. Integration, in this context, simply means the deliberate joining together of “inputs, organization and delivery of particular functions” to increase efficiency and access to health services ^8^. Stakeholders and scholars agree that more research is needed on how to effectively integrate PPFP in low- and middle-income countries ^9^. A Cochrane review of primary health service integration found that most studies with specific reference to family planning involved efforts to add family planning to HIV services ^8^. This review found limited evidence that adding family planning to other services leads to greater contraceptive use, and another recent review argued for more evaluation research ^10^. A third systematic review concluded that there was insufficient evidence, particularly about models for implementing PPFP programs ^11^. Although there is not conclusive evidence on the efficiency of family planning integration, evidence is mounting on the benefits of using every contact with pregnant and postpartum women to offer FP counseling and services ^12^. We also note that evaluations of integration focus on utilization and coverage ^11, 12^, not comprehensive assessments of quality; however client and provider perceptions of integration of services may well identify factors also associated with quality of care, such as waiting time or privacy.

Definitional issues cloud the integration literature, with some researchers focused on integration of services at the point of care and others focused on integration of national programs to channel resources and technical support for service delivery. When national programs are integrated (or ‘horizontal’ integration), the health system supports integrated services with planning, monitoring, supervision, and at times integrated supply chains, among other provisions ^13^. There is no evidence (yet) if service integration at scale requires program integration. Integrated services are assumed to be more efficient, in particular from the client’s perspective, but also for the health system ^14^. However, analysis of costs show wide variations in service integration between smaller facilities versus larger ones ^14^. From the provider’s perspective, there are concerns of added workload, which are somewhat balanced with increased job satisfaction ^15^.

Capitalizing on programmatic experience from PPFP programs in over 20 countries, we designed a study to assess the integration of PPFP with maternal, newborn, and child health (MNCH) services used by pregnant and postpartum women. Understanding factors affecting success or failure of integrated service delivery in different settings, i.e. increased adoption of multiple services with no decrease in quality, will assist health program managers to plan PPFP programs and focus their service integration efforts where they are most needed.

## Methods

This paper presents findings from one component of a descriptive evaluation of PPFP service integration conducted in India (May–June 2014) and Kenya (June-July 2014). This borader cross-sectional, mixed-methods study ^16^ examined how PPFP services are provided in order to identify factors enabling and limiting integration of PPFP with MNCH services in different settings.

### Study setting

We selected India and Kenya as study sites because of their long-running PPFP programs, which provided us a unique opportunity to evaluate provider and client experiences from different PPFP program models. The study was conducted in two of the 29 states in India (Jharkhand and Bihar), and two of the 47 counties in Kenya. (Embu and Siaya).

PPFP integration programs began in five states in India in 2010 and provided support for introducing PPFP counseling during antenatal care (ANC) and reorganizing intrapartum and immediate postpartum care to offer postpartum intrauterine device (PPIUD) services immediately after birth. This integration of services built upon the rise in institutional deliveries following the *Janani Suraksha Yojana* (JSY) incentive scheme established in 2005. Jharkhand was among the first five states to initiate PPFP services. Hospital providers received both a 3-day training on clinical services for PPIUD as well as a 2-day training in PPFP counseling. Hospitals also received tools and job aids to support counseling, services and data management, and program staff offered supportive supervisions visits jointly with health authorities. Programs replicated the same approach as in Jharkhand in Bihar, India, starting in 2012, but added community outreach and messaging on birth spacing and PPFP during home visits through a 1-day training of Accredited Social Health Activists (ASHAs) and auxiliary nurse midwives on family planning methods and key PPFP messages. Subsequent to experiences in the first states, PPFP continued to expand across India. Furthermore, the government created a dedicated PPFP counselor position and opened an initial 1,300 posts. The 2013 reproductive, maternal, newborn and child and adolescent health (RMNCH+A) strategy incorporated PPFP to make services available across India, but renamed the counselors as RMNCH+A counselors.

In Kenya, PPFP programs began on a small scale in Embu from 2006 to 2010 as part of a pilot study, and then through a Ministry of Health sanctioned training on integrated maternal and newborn and family planning training. This training consisted of an orientation on PPFP for providers in facilities with a high volume of deliveries, followed by a 5-day clinical training in PPIUD and infection prevention for nurse-midwives. Community health workers also received a 2-day orientation on PPFP. Program staff worked with county officials to offer supportive supervision and to identify PPFP “champions” to support postnatal care and PPFP. Meanwhile at national level, the 2009–2015 *National Reproductive Health Strategy* included the integration of family planning into other reproductive health services as a key approach for improving access to comprehensive reproductive health services. The *National Family Planning Guidelines for Service Providers 2010* specifies the postpartum timing for initiating various contraceptive methods. In 2012, a program in Siaya, Kenya, used a multidisciplinary approach to demonstrate integration of family planning and maternal, infant, young child nutrition (FP/MIYCN) into antenatal, intrapartum, postnatal, and early childhood care services at facility- and community-levels. A baseline assessment informed this demonstration project and the development of materials, followed by capacity building of facility providers, community health volunteers on how to counsel on family planning and nutrition, integrate FP/MIYCN in all service delivery areas (maternity, maternal and child health/family planning, outpatient department) and manage program data. Bondo subcounty- (formally referred to as a district) was the only subcounty- out of six in Siaya where PPFP and nutrition services had been integrated thus through the time of the study. Unlike the other three locations, the Bondo facilities did not offer PPIUD as an option immediately after birth. At the sub-county hospital, FP/MIYCN training took place onsite for three days along with a one-day whole-site orientation, while health center providers underwent five days of training with clinical practice.

### Sampling and data collection

In consultation with local health authorities, we purposively selected 15 health facilities in the two countries: six public health facilities from Bondo subcounty in Kenya and three facilities each from Embu county in Kenya, and Jharkhand and Bihar states in India. In India, the facilities were chosen based on health officials’ assessment of high performance, given the study objective to learn about what works to integrate PPFP. In Embu, Kenya, the health facilities were selected based on both performance and ease of access. There were six public facilities included in Bondo to capture all facility types participating in the nutrition and PPFP integration program. We published a separate manuscript on the family planning and nutrition integration experience from Bondo subcounty ^17^. Data collection methods in this study included a client flow analysis (Dataset 1, published elsewhere ^18, 19^); surveys of MNCH clients (Dataset 2) and providers (Dataset 3), and in-depth interviews with local authorities, facility management and health care providers. A convenience sampling approach was used for provider surveys and interviews. The number of participants at each facility was determined by the number of PPFP providers expected to be on duty, not a sample size/power calculation or content saturation. 

The study teams obtained letters of support from state- or county-level officials, alerted facilities ahead of arrival, then met with the person in charge for briefing and identification of providers available at the facility. Providers on duty on the day of interviews were approached for interviews or surveys. A study information sheet was posted at each facility and provided to in-charge and anyone else upon request.

MNCH service provider and client surveys were conducted at all 15 health facilities. At each facility, the number of providers surveyed using the structured questionnaire varied between one and four depending on the number of clinical staff at a facility and types of services provided. We collected data on the proportion of consultations that include PPFP counseling as well as their perceptions on PPFP services they provided.

With regard to clients, we surveyed a sample of pregnant women and mothers of children under age 2 years attending maternal or child health services to determine whether they received PPFP counseling during their visit on the day of the survey. For the client survey, the number of respondents sampled was designed to provide a study power of 80% to estimate ±10% with 95% confidence, in the estimation of the proportion of clients receiving integrated PPFP services. Clients responded to questions about wait time before consultation with a service provider, privacy during consultations (audio and visual), comfort discussing private/sensitive health matters, perceptions of service providers’ behavior towards them, and the availability of medications and FP commodities. Surveys were conducted using tablets loaded with CommCare software (CommCare v.2.11.0, Dimagi Inc.); it took 1–3 days to complete the surveys at each health facility. In India, service provider and client surveys were conducted in either Hindi or English depending on a respondent’s preference. In Kenya, all service provider surveys were conducted in English whereas client surveys were conducted in English, Swahili, or Luo, depending on the respondent’s preference. Surveys are available on Open Science Framework ^20^.

Client flow analysis was conducted at two facilities in each Indian state and in three facilities in each county in Kenya; five facilities with low client loads were excluded. Client flow tools were modified from a tool used previously to assess integration of HIV and sexual and reproductive health services ^21^. On days of data collection, research assistants approached all women aged 18 years or older who were at the health facility seeking MNCH services (ANC, postnatal, well-, or sick-child care), screened those who were willing to participate and consented those who met study eligibility criteria. Those who were pregnant and/or had a child under 2 years old and consented to participate were asked to carry a client-flow form which was completed by service providers at various service delivery points during the visit. Women in active labor were excluded since we could not expect them to keep track of providers caring for them and the form during childbirth. The client-flow form allowed up to five healthcare workers to check off services provided on a standard list of possible services and note any referral recommendations. The client-flow forms were collected at the point where each client exited the health facility.

Semi-structured, key informant interviews were conducted with local health authorities (district or sub-county officials), facility management and service providers to understand perceived benefits of PPFP integration, challenges in implementing integrated services, and the institutionalization of integrated services in the health system. In India, experienced qualitative interviewers from the International Institute of Health Management Research (IIHMR) conducted interviews. In Kenya, two independent consultants were hired and trained to conduct interviews. Key informants were selected on the basis of their involvement in supervising MNCH and PPFP services in the selected facilities. Each service provider was interviewed only once either as a facility health in-charge or about the PPFP services they provided. Providers were asked which service delivery unit they worked in (ANC, labor and delivery, postnatal care and/or child health). They were also asked to pick one of those units which they spend more time in and to answer subsequent questions about their work in that unit. At each site, qualitative researchers conducted interviews in a single day using structured interview guides designed specifically for each type of respondent: key informant, in-charge, or provider. Key informants and in-charges had to have a role in overseeing MNCH services in the facility including integration of PPFP. Service provider interviews were conducted in either Hindi or English in India, and solely in English in Kenya. Interviews lasted between 30 and 60 minutes and were conducted in private. While the study team discussed the concept of saturation with data collectors, they completed the number of interviews assigned, with no overlap between quantitative survey and qualitative interview respondents. All interviews were recorded, transcribed, and, if necessary, translated to English prior to analysis.

A total of 215 MNCH clients and 39 health care providers were surveyed, and 10 key informants and 33 facility informants interviewed, across the four study sites ( Table 1).

**Table 1.** Description of sample population by source location and data collection approach.

Site			Type of health facility	Interviews			Surveys		Client flow	*Subtotal* *by site*
				Key informants	Facility in- charges	Service providers	Service providers	Clients	Client flow	
**India**	**Jharkhand**	**1**	Hospital	1	2	4	4	20	290	623
		**2**	Hospital	1	2	4	4	15	252	
		**3**	Hospital	1	---	---	4	19	---	
	**Bihar**	**1**	Hospital	1	---	---	4	19	---	819
		**2**	Hospital	---	2	---	4	16	435	
		**3**	Hospital	1	1	---	4	15	317	
**Kenya**	**Embu**	**1**	Hospital	1	2	1	2	30	342	538
		**2**	Health Center	---	1	1	2	5	111	
		**3**	Health Center	---	1	1	2	5	31	
	**Bondo**	**1**	Hospital	4	2	---	5	36	228	475
		**2**	Health Center	---	1	1	1	7	66	
		**3**	Health Center	---	1	1	1	6	---	
		**4**	Health Center	---	---	1 *	1	7	86	
		**5**	Health Center	---	---	1	1	9	---	
		**6**	Health Center	---	2 *	1 *	---	6	---	
***Subtotal by tool***				10	17	16	39	215	2,158	**2,455**

* Interviews excluded from analysis after facilities sorted by level of integration (see Table 2)

### Data management and analysis

***Quantitative data analysis.*** Research assistants entered quantitative data from client and provider surveys into REDCap, version v6.0.1., a web-based software for data capture and management. Descriptive analysis was conducted using SPSS Statistics (version 22) and Stata (version 12). The investigators carried out frequency distributions and cross-tabulations and reported the outputs in percentages. No statistical testing was done.

***FP integration and qualitative data analysis.*** We analyzed qualitative data using a grounded theory approach. Interview scripts were read iteratively and open/conceptual codes generated. We conducted the FP integration analysis in three phases. The first phase comprised of four steps, as follows: Step 1: quantitative data from client surveys, health worker surveys, and client-flow analysis by facility were collated using Microsoft Excel. We excluded two facilities from Kenya where we did not collect client flow data because they had fewer than five client respondents per service area. Step 2: three research team members separately analyzed the collated client and provider survey data to generate independent assessments of the level of PPFP integration within maternal and child health services, to develop an approach similar to previous studies ^22, 23^. However, wide variation in levels of integration across service areas at many facilities, made it difficult to come to agreement about the overall level of integration in each facility. To address disagreements between the independent assessment of level of integration, we further disaggregated data by service area within each facility (ANC, Labor and Delivery, PNC, Child Health) and jointly analyzed the level of PPFP integration within each service area of each facility. In only two facilities were women having given birth in that visit interviewed. For each facility, analysis included the number of clients interviewed at the unit they initially sought care from (ANC, PNC or child health), and the percentage that indicated PPFP was discussed. Step 3: the number of providers surveyed, and their responses to the question as to how many among the last 10 clients seen in their ‘primary’ unit to whom they provided PPFP counseling or services (range 0–10). Step 4: client flow data were considered, specifically the number of clients who indicated that the purpose of the visit was ANC, labor and delivery, PNC or immunization/child health, as well as the number where the provider ticked having offered FP counseling or provision of a method. Box 1 provides further details about the specific survey used for this analysis and explains how the three researchers compiled the data for analysis.

**Box 1. Survey questions and data compilation for analysis of facility level of integration**

**Clients**

Analysis of the client data explored the primary reason for the visit:

“I’d like to ask you a few questions about your visit to this facility today. What was the primary reason for your visit?” Response options included: 1. Antenatal care, 2. Intrapartum/Labor room, 3. Postnatal/ Postpartum, 4. Child welfare/ Pediatrics, 5. Family planning, 6. Other, 98. Don’t know, 99. No response

Later in the questionnaire, clients were asked: “Now I’d like to talk to you about the main reason for your visit today. During your visit to ( *automatically populated with primary reason from question above*), did you discuss family planning?” with yes/no response options. This was the question used to assess level of integration, after disaggregation of clients according to whether they primarily visited for ANC, PNC, child health or labor and delivery.

**Providers**

Providers often work in multiple service areas in a facility, but were asked to select one area (from a list of ANC, Intrapartum/Labor room, Postnatal/Postpartum care, Child welfare/Pediatrics) that would become the subject of subsequent questions.

Later in the survey, providers were asked to quantify these responses with: “I would like to ask you a couple of questions about the last ten clients you have seen who were [pregnant / recently gave birth / or had given birth in the last two years]. Of those ten women, to how many did you either provide family planning counseling or a family planning method?”

**Client flow**

Data were stratified by the same primary reason for visit as above, and we captured a cell count of the number of clients visiting the facility for that reason during data collection as a denominator. Then the count of all clients whose form included a mention of FP counseling or FP services for each of the primary visit reasons strata were included in the numerator.

**Data compilation and review**

We created a spreadsheet with rows for each health facilities and columns for each type of service (ANC, PNC, child health, or labor and delivery). We first included a count of all data points for each (client, provider surveys, client flow data) as a denominator. Then, for each facility and type of service, we populated the number of clients reporting FP discussion, the number among last 10 clients seen that a provider reported offering FP counseling, and the clients whose forms included FP integration, as numerators. For providers, this resulted in a series of numbers from 0-10. The average of those numbers was used. For the other data, simple percentages were calculated.

Similar to previous studies ^22, 23^, we used an ordinal scoring system to characterize the level of service integration, where 1 = low levels of integration (0-29% of MNCH client visits included PPFP and survey data suggested little integration), 2 = moderate levels of integration (30–59% of visits included PPFP and survey data suggested some integration), and 3 = high levels of integration (60–100% of visits included PPFP and survey data suggested integration). In cases where there was not concordance between client flow and interview data, we relied on the client flow data as a more robust source. Independent rankings were then reviewed by all three researchers, although at that point the analysis relied on scores so the discussion focused more on whether there was sufficient data to determine an integration level. We set a minimum of 5 clients responses. There was insufficient data to consistently analyze level of PPFP integration in PNC and child health services. We thus used the levels of integration in ANC to disaggregate health facilities with high and moderate levels of integration and grouped these as well integrated (shown in bold text in Table 2) and those with low-levels of integration we termed poorly integrated (shown in non-bold text in Table 2). The decision to group the middle tier of integration as “well integrated” was made on the assumption that this integration was unlikely to be by chance, but as a result of the program having some effect.

**Table 2.** Levels of FP integration into different services areas by facility and country. KO03 and KO06 excluded because insufficient data for all units. Text indicates where on broad range of level of integration (30–100%). If there are two notations, it indicates differences in client flow and client survey data, where at least 5 clients were surveyed about that service area. Bold text indicates well-integrated care, non-bold indicates poorly integrated care.

	Facility code	Antenatal care *	Labor & delivery	Postnatal care	Child health
India	IJ01	**High**	*No data*	**High**	**Med-high**
	IJ02	**High**	*No data*	**High**	Low
	IJ03	**High**	*No data*	*No data*	*Insufficient data*
	IB01	**High**	**High**	*No data*	*No data*
	IB02	**Med**	**High**	Low	Low
	IB03	Low	*No data*	*Insufficient data*	Low
Kenya	KE01	Low	*No data*	Low	Low
	KE02	Low	*No data*	*Insufficient data*	Low
	KE03	Low	*No data*	*No data*	Low
	KO01	Low	*No data*	**Med**	Low
	KO02	**Med**	*No data*	**High**	**Med**
	KO04	**High**	*No data*	**High**	**Med-low**
	KO05	*Insufficient* *data*	*No data*	*No data*	**High**

*Unlike the other service areas, integration of FP in antenatal care involves only FP information, counseling, possibly condoms and referrals. In other service areas, there should also be provision of a contraceptive method or intra-facility referral for provision.

The second phase of analysis involved reviewing the semi-structured interview transcripts to identify factors enabling and limiting integration of PPFP services at each facility and coded the text using a combination of thematic and free-coding in ATLAS.ti 7.5 (Scientific Software Development GmbH, Berlin, Germany). We reviewed qualitative findings across sites to identify characteristics common to well-integrated facilities separately from those that were poorly integrated.

In the third phase, we analyzed antenatal care client reports of privacy, interactions with staff, and waiting times to compare experiences at well-integrated and poorly integrated facilities by calculating percentages per site. We did not use the same approach for postnatal care or child health clients because of the small numbers of these respondents. To synthesize findings, we triangulated analyses described above, and examined trends in client, provider and management experiences at well-integrated and poorly integrated facilities in each study county, country, and overall.

### Ethical considerations

The study was approved by the Johns Hopkins School of Public Health institution review board (No 5517), IIHMR’s Institutional Review Board in India, and the Kenya Medical Research Institute (KEMRI) Ethical Review Committee in Kenya. All participants provided oral informed consent, in accordance with approved research protocols, in order to avoid collecting personal identifiers for study participants.

## Results

This study assessed the extent to which PPFP is integrated in MNCH services and identified factors affecting service delivery in four distinct public sector settings, two in Kenya and two in India. Kenyan sites included 2 hospitals (1 in Embu, 1 in Bondo) and 7 health centers (2 in Embu, 5 in Bondo), while all six facilities selected in India were hospitals. All facilities had been providing PPFP services for at least two years. Clients’ mean (SD) age was 24.4 (4.8) years, and the majority were married (100% in India; 80% in Bondo, and 85% in Embu). Clients’ level of education varied greatly; 60% of clients in India had completed secondary school or higher, compared to 80% of clients in Embu, Kenya and 20% of clients in Bondo, Kenya. Data from provider and client exit interviews are available on Open Science Framework ^20^.

### Phase 1: Levels of service integration

Table 2 shows the level of PPFP integration across groupings of MNCH services at each facility: 1) within maternal health services (ANC or labor/delivery) and 2) within child health services (postnatal and well- or sick- child care services). Of the 13 facilities examined, only two (one from Kenya and one from India) were deemed to have a high level of integration in both groupings of MNCH services. There were six facilities (three from Kenya and three from India) that demonstrated a high level of service integration in either maternal health or child health services.

Because some determinants of integration may be a function of contextual factors, it is reasonable to expect facilities within the same national or subnational health system to be more similar to each other than to facilities in other areas. Our analysis revealed some similarities in levels of integration by facility type and service area, but scores were not consistent enough to draw strong conclusions about overall levels of integration achieved in all study sites. For example, in Jharkhand, India, PPFP appeared to be well integrated in maternal health services, but only one of three facilities had integrated PPFP in child health services. Health centers in Bondo, Kenya, integrated PPFP in child health services relatively well and performed better than the larger health facility that supports them (the sub-county hospital). Health facilities (a hospital and two health centers) in Embu, Kenya, showed the lowest degree of PPFP integration, while facilities in Bihar, India, showed the most variation between well- and poorly integrated services. Analysis was also limited by imbalance in the number of clients surveyed at each facility. It is worth noting that the number of clients surveyed at well-integrated facilities in India was about four times greater than at poorly integrated facilities.

### Phase 2: Factors influencing success of service integration

Although levels of integration varied by service area and health facility, common themes were expressed in semi-structured interviews at both well-integrated and poorly integrated facilities. The themes were related to the importance of: (1) dedicating appropriate space for PPFP services, (2) considering health workforce composition, capacity, and motivation, and (3) ensuring consistent and affordable supply of a range of contraceptive methods. The influence of these factors on the level of PPFP integration were also evident in findings from provider and client surveys. 

***Appropriate space for PPFP services.*** Space provisions, including appropriate spaces for PPIUD insertion, were frequently mentioned as factors enabling PPFP integration. Highly integrated facilities were more likely to have dedicated spaces for PPFP counseling, which may or may not be separate rooms but provide privacy and space for client and counselor. However, our findings also show that a dedicated space for PPFP services alone is not sufficient for successful integration of services, and that not all dedicated spaces adequately meet provider needs:

“ *…the original plan did not have the integration aspect in mind. So the current existing room could be very small, like the one we are seated in... Now that [PPFP] is coming in, you may need an extra tray table for demonstration. You need a couch for examination and, if that space is not there, then you find it can be a challenge until the staff can generally say there is nothing we can do.”* [Provider, Bondo, well-integrated]

In addition to not having dedicated spaces for PPFP services, poorly integrated facilities were characterized by chaotic environments:

*“It is in different room therefore they face problem in inserting IUCD…[T]here is no special room [for inserting PPIUD]. And most importantly, I want our counseling room to be improved. I mean when a client visits my room after meeting the doctor, then she must feel at ease here and can talk to us in relaxed state of mind...And here, also, you hear continuous noise to the point where, if someone tries to speak, nobody can listen clearly. This place is just besides the outpatient department; therefore, there is a continuous rush over here.”* [Counselor, Bihar poorly integrated]

Additional infrastructure issues, such as the physical layout of a facility, may pose challenges to effective integration of PPFP services. Data collection team members shared that one Bihar facility layout required clients to navigate complicated pathways to various units; this facility was independently ranked as being poorly integrated.

**Table 3.** Antenatal care clients report on privacy, interactions with staff, and waiting time.

Characteristic		Kenya			India		
		Well integrated (n = 14)	Poorly integrated (n = 71)	Total (n = 85)	Well integrated (n = 66)	Poorly integrated (n = 15)	Total ^a^ (n = 81)
**Visual &** **auditory** **privacy**	**Not a** **problem**	13 (92.9%)	62 (87.3%)	75 (88.2%)	20 (30.3%)	12 (80.0%)	32 (39.5%)
	**Minor/major** **problem**	1 (7.1%)	9 (12.7)	10 (11.8%)	46 (69.7%)	3 (20.0%)	49 (60.5%)
**Comfort** **discussing** **private or** **sensitive** **health** **concerns**	**Not a** **problem**	12 (85.7%)	60 (84.5%)	72 (84.7%)	25 (37.9%)	11 (73.3%)	36 (44.4%)
	**Minor/major** **problem**	2 (14.3%)	11 (15.5%)	13 (15.3%)	41 (62.1%)	4 (26.7%)	45 (55.6%)
**#Waiting** **time**	**Not a** **problem**	9 (64.3%)	44 (62.0%)	53 (62.4%)	22 (33.3%)	11 (73.3%)	33 (40.7%)
	**Minor/major** **problem**	5 (35.7%)	27 (38.0%)	32 (37.6%)	44 (66.7%)	4 (26.7%)	48 (59.3%)
**Perception** **of how staff** **treat clients**	**Not a** **problem**	13 (92.9%)	67 (94.4%)	80 (94.1%)	31 (47.0%)	12 (80.0%)	43 (53.1%)
	**Minor/major** **problem**	1 (7.1%)	4 (5.6%)	5 (5.9%)	35 (53.0%)	3 (20.0%)	38 (46.9%)

^a^ In total, 215 clients were interviewed as indicated in Table 1 and responded to a series of “statement” questions: minor problem; major problem, or no problem. These responses were cross-tabulated with the variable on the level of integration at ANC (poor vs. well). Based on earlier analysis and categorization of the integration, some health facilities ended up NOT having a level of ANC integration being assigned to them, due to “inadequacy of available information”. Correspondingly, the responses of clients in these respective health facilities were “dropped” during cross-tabulation. In Kenya, 26 records were dropped, while in India, 23 records were dropped, for a total of 49 records.

***Health workforce composition, capacity, and motivation.*** Having a dedicated, well-trained PPFP counselor on staff was another factor identified as contributing to successful integration of services, as it increases the likelihood that clients will have their PPFP needs addressed in the same visit. As an in-charge of a well-integrated Jharkhand facility explained, “ *We have the counselor here specifically for counseling, and she is able to give enough time.”* Well-integrated facilities were also more likely to have motivated staff willing and able to take on additional responsibilities. One Bihar provider at a well-integrated site noted, *“I have learned by hearing others. And I made my mind that [counseling] is my responsibility, and I have to do it.”*

Staffing and wait time are often linked in provider statements, but providers who are motivated to implement integrated services may portray the issue differently. For example, a service provider from a well-integrated facility noted:

*“[Integration] is good because everybody can provide the services, and the client does not need to wait for one person or go to a particular service provision place for the [family planning], and that has really helped in reducing the [loss] of clients, as well as reducing the time they take when they come for those services.”* [Provider, Bondo, well-integrated]

Poorly integrated facilities were often staffed by individuals either unable or unwilling to take on responsibilities beyond what they saw as their traditional job tasks. In many cases, the facilities ascribed this to shortages of health workers, especially in rural and remote areas. A nurse in a poorly integrated site in Bihar, India, explained that at the facility where she works, *“No one fulfills his/her responsibilities appropriately due to scarcity of staff.”* Additionally, service providers in Embu echoed this constraint, explaining that the integration of PPFP counseling has increased wait times for clients, generating complaints that negatively impact job satisfaction. As one Bondo In-charge explained, *“If the staffing improves, then I think the services will also improve more because sometimes someone may not have all time…like now you see [a family planning patient] is waiting for you…so sometimes one may be tempted to move faster than it should be and that may compromise the service.”*

A related but distinct issue around staffing relates to provider training and preparation to offer integrated services. As a manager in a poorly integrated facility in Embu, Kenya, explained, *“What we needed most was training of which we still need up to now. As you have heard, we had one person trained on PPIUD who now left, so we would like more people to be trained.”* Providers also saw training on PPFP counseling and service provision as a professional development opportunity and noted the integration of counseling in other service areas provided clients with opportunities for closer interaction with service providers, resulting in increased trust in the health system:

*“So things are going well here in district hospital. However, one portion, in particular, is weak from our side and [that] is counseling. Counseling, which is related to ANC period. Counseling is weaker; therefore, we are not able to effectively facilitate the decision-making of mothers visiting our facilities. Mothers who visit the facilities for delivery needed time for mind-making, for taking decisions. So if our counseling is improved, then our overall performance would be improved.”* [Key Informant, Jharkhand]

Although we only surveyed 17 ANC providers, responses found that higher proportions of providers in well-integrated facilities reported having knowledge and skills to provide both PPFP counseling and services, compared to providers in poorly integrated facilities.

***Consistent, affordable supply of contraceptive methods.*** Key informants and facility staff in Kenya and India highlighted the importance of access to and affordability of contraceptive commodities. A Jharkhand facility in-charge in a well-integrated facility also mentioned the role of educational materials, recalling earlier times before a counsellor joined the staff: “ *We were not able to tell about the methods, and, also, we didn’t have this kit so we were not sure whether [the mother] understood about the methods or not.”* Because the type of family planning supplies needed vary across service areas, it was challenging to assess differences in commodity availability within our relatively small sample of well- and poorly integrated facilities.

At some facilities, staff reported that increased demand for contraceptives generated by integrating services initially resulted in stock-outs, but, over time, they were able to establish systems to address this issue. Others reported occasional stock-outs or little information on availability of commodities. Facilities with mid to high levels of PPFP integration tended to have a well-functioning supply chain management system while some poorly integrated facilities did not monitor their supply levels. As a government official with oversight responsibilities for a poorly integrated facility in Bihar explained, *“Yes, there have been [stock-outs] sometimes. […] I cannot tell you much about this, frankly speaking as many times our staff could not check these things.”*

In some facilities, commodities may be available, but repeated travel to the facility remained a barrier to the continuum of care. For example, a manager in a poorly integrated facility in in Embu, Kenya noted *“…depending on where she is coming from and her status she may not be able to afford all these journeys, so [PPIUD] saves time for everybody.”*

***Context matters: Additional factors affecting the success of integration efforts.*** In addition to the issues discussed above, two context-specific themes were raised throughout semi-structured interviews in Jharkhand and Bihar, India, as major factors affecting the success of integration efforts: provider-client power dynamics and demand-side financing programs.

In both Bihar and Jharkhand, providers articulated that they think some challenges to PPFP uptake are due to what they perceive as clients’ religious beliefs and education levels. In Jharkhand, a head nurse described the clients as *“backwards,” “uncivilized,”* and *“uneducated.”* In Bihar, providers counsel religious clients that not spacing and caring for kids is a sin against God. Providers in both Jharkhand and Bihar opined that Muslim women refuse PPFP because of their cultural beliefs, specifically citing that if a woman is operated on, she will not be placed for burial after death. Additionally, providers highlighted that how they counsel a woman is often based on the number of children the woman already has rather than the woman's expressed preferences or needs. As one counselor in Bihar (well-integrated facility) explained, *“We try to motivate those for sterilization who have two children.”* While these attitudes are independent from integration efforts, integration implies an increase in number of providers discussing family planning with clients and that these providers need additional guidance on the rights of family planning clients to make voluntary and autonomous decisions about contraception.

In addition, many key informants and facility staff in both states of India mentioned the effects of financial incentives, specifically the JSY conditional cash transfer program, on PPFP uptake. Incentives are frequently used in India to assist in motivating clients as well as health workers. The role of incentives varied across Bihar and Jharkhand, but respondents in all sites agreed that they are very important. In Jharkhand, an in-charge of a well-integrated facility highlights how the incentives have shifted community norms and helped motivate other clients to come: *“we keep on telling one thing, so people understand and after that they tell others also. Then they are also getting money [JSY], so people come.”* However, whether these incentives are in the best interest of the client is cloudy.

Semi-structured interviews conducted in Kenya did not yield as rich descriptions of factors affecting the success of service integration efforts as interviews conducted in India. However, one provider in Bondo expressed a sense of teamwork with the sub-county health management team:

*“So all the commodities, the infrastructure, the equipment, the furniture and, of course, that consistent support supervision. Even as we make report, we forward [the report to] them and they are able to share with us. This…is the support in itself because, maybe during that preparation of that report, there is a glaring error that [is noticed] and I missed it. So the sub-county record person during that editing time, just a phone call, they have done this [asked about data, and corrected an error]. [Provider, Bondo, well-integrated]*

This teamwork approach was also expressed by providers resolving commodity issues or coordinating with mobile teams to offer female sterilization at their facility.

### Phase 3: Client experiences

All ANC clients surveyed were asked about privacy concerns if PPFP was discussed during their visit. A greater proportion of clients in Kenya than in India, reported acceptable levels of privacy (88.2% in Kenya versus 39.5% in India) and feeling comfortable discussing private/sensitive health concerns (84.7% in Kenya versus 45% in India). When comparing well-integrated and poorly integrated facilities in Kenya, the vast majority of clients at facilities in both categories reported that visual and auditory privacy were not a problem (92.7% at well-integrated facilities versus 87.3% at poorly integrated facilities). In India, by contrast, stark differences emerged between well- and poorly integrated facilities. At well-integrated facilities, the majority (69.7%) of clients reported privacy as a problem, compared with 20.0% at poorly integrated facilities, as shown in Table 3. In all sites, having concerns about visual/auditory privacy was correlated with concerns about discussing private/sensitive health concerns.

In the qualitative analysis, some providers reported that long client wait time was a disadvantage of integrating PPFP counseling with MNCH services. This concern was not highlighted as consistently by ANC clients in Kenya, where only 35.7% of clients in well-integrated facilities and 38.0% in poorly integrated facilities reported wait time as a problem. However, in India, the majority of ANC clients (66.7%) in highly integrated facilities reported wait time as a problem as compared with only 26.7% of ANC clients at poorly integrated facilities, as shown in Table 3. We note that all well-integrated facilities in Kenya were health centers, whereas all sampled India facilities were hospitals.

## Discussion

Our findings suggest that programs to encourage integration take hold unevenly across facilities, and we have sought to better understand how to encourage greater effective integration of services. Despite very different contexts, the factors identified as contributors or barriers to successful integration of PPFP in MNCH services were remarkably similar in both India and Kenya. Common themes identified across settings related to (1) dedicating appropriate space for PPFP services, (2) considering health workforce composition, capacity, and motivation, and (3) ensuring consistent and affordable supply of a range of contraceptive methods.

These findings are consistent with emerging evidence from other settings. Integration of health services is often assumed to be an efficient strategy for increasing access to care and a “diagonal approach to building primary healthcare” has been espoused ^24^. Technical experts generally agree that optimal delivery of PPFP requires: taking advantage of existing contact points; direct provision of PPFP counseling or services during maternal, newborn, and child health (MNCH) consultations; or facilitated referrals to a co-located family planning provider ^6, 25^. Without interventions to coordinate the care provided, however, co-locating services in a facility has not been shown to increase the use of multiple services ^26^. For example, in Uttar Pradesh, India, only a minority of antenatal and postnatal care clients receive advice about PPFP or birth spacing; as a result, unmet need for family planning has not decreased ^27, 28^. Further, a systematic review of integrated HIV and family planning services noted that facilitated referrals between co-located services were difficult to implement without a solid health system foundation, and weak fidelity to the designed service integration intervention may have led to lower than expected family planning uptake ^29^. Changing the organization of services for the purposes of integration requires one or more interventions, such as training, new tools, supervision, and performance improvement. At the same time, fully integrating PPFP services across the continuum of care may have trade-offs for resource allocation and attention to other aspects of service quality ^8^.

Respondents from well-integrated Kenyan facilities cited provider competence and availability of FP commodities as facilitators, whereas poorly integrated facility respondents cited inadequate space, staff shortages, workload, end-user costs, and commodity stock-outs as barriers. While commodities are necessary for any service, integrated or not, shortages act as a disincentive to integrate services as that may exacerbate the problem of stockouts. Respondents from well-integrated facilities frequently complained about lack of space, yet they seemed to work around this challenge, indicating factors beyond space are critical for successful integration. Providers from well-integrated Indian facilities described having a room for the dedicated counselor, motivation to provide client-centered care, providers who believe in PPFP benefits, acceptance of workload, training for staff, continuous supply of FP commodities, and incentives for PPIUD insertions as favorable. Service providers in poorly integrated facilities in India said not all client costs were covered and reported low motivation (e.g., PPFP counseling was not their job), although underlying reasons for variances in provider motivation within the same system could not be ascertained in this study. Other researchers have noted that integration of physical and human resources (‘structural integration’) is insufficient to ensure that clients consistently receive integrated services (‘functional integration’) ^30^.

Within geographic locality, program support was generally similar, yet the level of integration of PPFP within maternal health services or child health services varied. Of note, one Jharkhand facility has gone beyond initial program interventions to integrate PPFP strongly into child health services. Facilities in Bihar had the most variation in level of integration. In that state, qualitative results suggested that providers were much more motivated by incentives.

This findings of this study are in line with the findings of Mayhew *et al*., that there is considerable heterogeneity in levels of integration across their study sites in Africa, including Kenya ^30^. The emphasis on incentives in Bihar could also imply that counseling is more systematic for women in labor and delivery and the immediate postpartum period, with potential missed opportunities in ANC. We cannot reliably report on the level of service integration or how consistently PPFP is integrated in a childbirth visit from our data. Nevertheless, quality PPFP should begin with ANC as women often need time to consult and consider their contraceptive method of choice ^31^. The opportunity to do so was more evident in our sample of facilities in Jharkhand. The ethical issues associated with incentives have been noted in the literature ^32^.

In Embu, Kenya, the level of service integration was unsurprisingly lower than in other sites, most likely due to the short duration of project support and the elapsed time between end of project and this assessment—about 5 years. Furthermore, attrition of trained providers affected service integration in those sites, particularly the smaller facilities. Sweeney and colleagues, in their study of HIV, sexual and reproductive health and postnatal care integration, reported on the slow pace of change to integrate health services ^33^. This implies that a short intervention period to effect change in service integration may not bring about lasting change as was seen in Embu. In comparison, integration was stronger in Bondo health centers, which have fewer staff who tend to provide a greater number of services, as compared to hospitals which have more staff who tend to work in distinct units providing a smaller number of services. Integration of services was also stronger in postnatal or child health services, as compared to ANC or labor and delivery services, which may reflect the ease of service integration when the program emphasizes integrating PPFP into infant and young child services.

Most of the poorly integrated facilities had staff who reported high workloads, lack of skills to insert PPIUDs, frequent stock-outs of family planning commodities, and client privacy challenges. On the other hand, providers in well-integrated facilities in India and Kenya reported greater commitment to high-quality services for clients. This contrasts with the study by Church *et al*., which found that providers rarely saw personal benefits of integrated care HIV and reproductive health care, perceiving benefits mostly for clients ^34^. Clients seeking ANC services in well-integrated facilities in Kenya did not report concerns with wait time or privacy. In well-integrated facilities in India, however, these factors, along with lack of concern for client autonomy and method choices, were reported as minor or major problems. Our relatively small, descriptive client sample precludes drawing firm conclusions about client reports of privacy concerns between well- and poorly-integrated facilities, but this merits further exploration. Other studies in India have found that providers negatively judge clients’ education, family planning needs, and ability to understand contraceptive options, thereby imposing unnecessary barriers to acquiring family planning methods ^35^. This study suggests that integrating FP services within other services suffers the same provider bias issue. Nevertheless, provider reports of barriers to integration in this study are not conclusive given that time-motion studies show that health workers exaggerate time needed for a given health intervention ^30^.

When reviewed through the lens of poorly versus well-integrated services, analysis of client responses on different aspects of service quality (privacy, comfort, wait time, staff treatment of clients) provided interesting insights. For example, a higher proportion of clients in well-integrated facilities in India, compared to those in poorly integrated facilities, reported problems with various aspects of services, which may seem striking. However, clients from poorly integrated facilities were less likely to be exposed to PPFP services, which are often considered to be a sensitive issue in our Indian study sites. Similarly, clients from well-integrated facilities were more likely to receive PPFP services, which may have led to increased concerns of privacy and discomfort discussing sensitive issues. Greater frequency of concerns about a lack of privacy and comfort discussing PPFP, as reported by clients in India compared with Kenya, may be due to perceived cultural sensitivity about family planning overall, which have been reported elsewhere ^36^.

In India, the finding that wait times were more likely to be perceived as a problem in well-integrated facilities is also not surprising given high client volumes in these facilities. This finding is also consistent with case studies of integration in sub-Saharan Africa ^37^. Of concern in the Indian sites is the disproportionate number of clients reporting mistreatment by staff as a problem in well-integrated facilities, compared with poorly integrated facilities. Furthermore, the influence of monetary incentives may induce certain providers to apply more pressure on women to adopt a contraceptive method, which would amount to coercion and against the principle of informed choice when providing family planning services.

As an exploratory study attempting to identify trends in health facility and service delivery characteristics across four distinctly different program settings, our analysis is not without limitations. Our analysis of levels of integration relies heavily on an assessment done through a client-flow and data collection activity that was not feasible to conduct with women attending facilities for childbirth so may not accurately represent levels of PPFP integration across all MNCH services. In addition, the 1-week duration of observation may not accurately represent levels of integration over time. We acknowledge that systematic observation of provider-client interactions in a clinic setting over a longer time period would probably have generated more robust data. Second, the number of facilities sampled in each location was small and precluded generating insights about the type or size of facility that may be more amenable to integrating services. The purposive sampling also implies that our results may not be generalizable. Third, as with all client and provider surveys conducted on-site at health facilities, there is a risk of courtesy bias. Fourth, our interview data focused heavily on ANC clients as opposed to immediate postpartum clients, as the study team felt it burdensome to ask women to participate in an hour-long interview immediately after giving birth. However, a substantial number of PNC clients were interviewed at the child health services, thus perspectives of postpartum women were included in our analysis and the use of a mixed method approach with data collection tools designed to explore many aspects of service provision allowed us to gather valuable insights in settings where there is limited evidence on effective strategies for service integration. We also recommend additional research on establishing measures of service integration that should be validated in randomly selected facilities.

Our findings highlight the complexities that must be considered in implementation and evaluation of health service integration. Policymakers contemplating increased integration should thus base their strategies on an understanding of the diversity in the health facilities they seek to affect. Policymakers also need new ways to measure whether integration of services is actually taking place as prescribed. Our findings showed that, even after program support to integrate services, actual levels of service integration varied greatly.

## Conclusions

Through triangulation of data from clients, providers and a client flow tool, our study sought to innovatively measure and qualitatively understand the level of PPFP integration with antenatal and child health services and factors that enabled or limited this integration at facilities that receive program support to integrate PPFP into maternal and child services. The factors identified were common across Kenya and India. Common themes emerged from interviews with providers and facility in-charges at both well- and poorly integrated facilities: issues influencing integration included allocation of physical space for PPFP service delivery, health workforce composition and capacity, and commodity availability, having staff to manage the combined services, client wait times, the organization of services to ensure that commodities are readily available, and as well as duration and nature of related program support. Service providers in well-integrated facilities are more likely to express motivation for client-centered, integrated care. Our analysis contributes to the limited literature on the integration of PPFP with maternal and child health services. The results are not clear cut but show the complexity and nuances of effectively implementing and scaling up integrated services. Better measures are needed to verify whether services are integrated as prescribed by national policies.

## Data availability

### Underlying data

Dryad: Data from: Postpartum family planning integration with maternal, newborn, and child health services: a cross-sectional analysis of client flow patterns in India and Kenya. https://doi.org/10.5061/dryad.11313 ^19^. The base dataset for client flow data is available in SAV, DTA and CSV formats. A codebook is available in DOCX format. Data were generated in a previous study ^18^.

Open Science Framework: PPFP Integration in India and Kenya. https://doi.org/10.17605/OSF.IO/5TFA7 ^20^. The following underlying data files are available:

- Provider interviews (Tool 3b_FINAL.csv) with codebook (Tool 3b_CODEBOOK_SEED_2.csv).
- Client exit interviews (Tool 5a_FINAL.csv) with codebook (Tool 5a_CODEBOOK_SEED_2.csv).

In addition, we collected, transcribed and translated, where appropriate, in-depth interviews (qualitative data). The study team feels it would be impossible to completely de-identify qualitative interviews. Furthermore, since the protocol approved by 3 ethical review committees (United States, India and Kenya) did not allow for sharing of qualitative data, we are not able to provide this dataset. If researchers would like to use the qualitative data, to either replicate our analysis or for other purposes, they may contact the corresponding author. They will be asked to sign a data use agreement prior to receiving any transcripts. We may also request that they obtain permission from the corresponding in-country IRBs.

### Extended data

Open Science Framework: PPFP Integration in India and Kenya. https://doi.org/10.17605/OSF.IO/5TFA7 ^20^. The following extended data files are available:

- Key Informant Interview Guide – Tool 1 (PPFP_Integration_IRB5517_Tool1-KI_IntGde_English_v3_2014May06.docx)
- Facility In-Charge Interview Guide (bilingual) – Tool 2 (PPFP_Integration_IRB5517_Tool2-Fac_InChg_IntGde_Bilingual_India_v2_2014May06.docx)
- Facility-Based Provider Interview Guide – Tool 3a (PPFP_Integration_IRB5517_Tool3a-Fac_Prov_IntGde_Bilingual_India_v2_2014May06.docx)
- India: Facility-Based Provider Survey – Tool 3b (PPFP_Integration_IRB5517_Tool3b-Fac_Prov_Survey_India_v2_2014May02.docx)
- Kenya: Facility-Based Provider Survey – Tool 3b (PPFP_Integration_IRB5517_Tool3b-Fac_Prov_Survey_Kenya_v2_2014Jun05.docx)
- India: Client Survey – Tool 5a (PPFP_Integration_IRB5517_Tool5a-Client_Survey_India_v2_2014May21.docx)
- Kenya, Luo: Client Survey – Tool 5a (PPFP_Integration_IRB5517_Tool5a-Client_Survey_Kenya_v2_2014Jun05_Luo_TRACKED.doc)
- Kenya, Swahili: Client Survey – Tool 5a (PPFP_Integration_IRB5517_Tool5a-Client_Survey_Kenya_v2_2014Jun05_Swahili_TRACKED.docx)
- Kenya, English: Client Survey – Tool 5a (PPFP_Integration_IRB5517_Tool5a-Client_Survey_Kenya_v2_2014Jun05_TRACKED.docx)
- Integration Client Flow Form – Tool 5b (PPFP_Integration_IRB5517_Tool5b-Client_Flow_ENG_2014May03.pdf)

Data are available under the terms of the Creative Commons Zero "No rights reserved" data waiver (CC0 1.0 Public domain dedication).

## References

[1] Stover J Ross J: Changes in the distribution of high-risk births associated with changes in contraceptive prevalence. *BMC Public Health.* 2013;13 Suppl 3:S4. 10.1186/1471-2458-13-S3-S4 24564577 PMC3847521

[2] Kozuki N Lee AC Silveira MF: The associations of birth intervals with small-for-gestational-age, preterm, and neonatal and infant mortality: a meta-analysis. *BMC Public Health.* 2013;13 Suppl 3:S3. 10.1186/1471-2458-13-S3-S3 24564484 PMC3847557

[3] Cleland J Conde-Agudelo A Peterson H: Contraception and health. *Lancet.* 2012;380(9837):149–56. 10.1016/S0140-6736(12)60609-6 22784533

[4] Moore Z Pfitzer A Gubin R: Missed opportunities for family planning: an analysis of pregnancy risk and contraceptive method use among postpartum women in 21 low- and middle-income countries. *Contraception.* 2015;92(1):31–9. 10.1016/j.contraception.2015.03.007 25769442

[5] Gaffield ME Egan S Temmerman M: It's about time: WHO and partners release programming strategies for postpartum family planning. *Glob Health Sci Pract.* 2014;2(1):4–9. 10.9745/GHSP-D-13-00156 25276558 PMC4168603

[6] World Health Organization (WHO), United States Agency for International Development (USAID), Maternal and Child Health Integrated Program (MCHIP): Programming strategies for postpartum family planning. World Health Organization,2013 Reference Source

[7] Vernon R: Meeting the family planning needs of postpartum women. *Stud Fam Plann.* 2009;40(3):235–45. 10.1111/j.1728-4465.2009.00206.x 19852413

[8] Dudley L Garner P: Strategies for integrating primary health services in low- and middle-income countries at the point of delivery. *Cochrane Database Syst Rev.* 2011; (7):Cd003318. 10.1002/14651858.CD003318.pub3 21735392 PMC6703668

[9] Ali M Seuc A Rahimi A: A global research agenda for family planning: results of an exercise for setting research priorities. *Bull World Health Organ.* 2014;92(2):93–8. 10.2471/BLT.13.122242 24623902 PMC3949533

[10] Kuhlmann AS Gavin L Galavotti C: The integration of family planning with other health services: a literature review. *Int Perspect Sex Reprod Health.* 2010;36(4):189–96. 10.1363/3618910 21245025

[11] Sonalkar S Mody S Gaffield ME: Outreach and integration programs to promote family planning in the extended postpartum period. *Int J Gynaecol Obstet.* 2014;124(3):193–7. 10.1016/j.ijgo.2013.09.021 24434229 PMC4040294

[12] Cleland J Shah IH Daniele M: Interventions to Improve Postpartum Family Planning in Low- and Middle-Income Countries: Program Implications and Research Priorities. *Stud Fam Plann.* 2015;46(4):423–41. 10.1111/j.1728-4465.2015.00041.x 26643491

[13] Atun R de Jongh TE Secci FV: Integration of priority population, health and nutrition interventions into health systems: systematic review. *BMC Public Health.* 2011;11:780. 10.1186/1471-2458-11-780 21985434 PMC3204262

[14] Shade SB Kevany S Onono M: Cost, cost-efficiency and cost-effectiveness of integrated family planning and HIV services. *AIDS.* 2013;27 Suppl 1:S87–92. 10.1097/QAD.0000000000000038 24088688

[15] Mutemwa R Mayhew S Colombini M: Experiences of health care providers with integrated HIV and reproductive health services in Kenya: a qualitative study. *BMC Health Serv Res.* 2013;13:18. 10.1186/1472-6963-13-18 23311431 PMC3599716

[16] Cresswell JA Plano Clark V: Designing and Conducting Mixed Methods research. Thousand Oaks, CA: Sage Publications;2007 Reference Source

[17] Cooper C Ogutu A Matiri E: Maximizing Opportunities: Family Planning and Maternal, Infant, and Young Child Nutrition Integration in Bondo Sub-County, Kenya. *Matern Child Health J.* 2017;21(10):1880–1889. 10.1007/s10995-017-2341-9 28766091 PMC5605598

[18] Mackenzie D Pfitzer A Maly C: Postpartum family planning integration with maternal, newborn and child health services: a cross-sectional analysis of client flow patterns in India and Kenya. *BMJ Open.* 2018;8(4):e018580. 10.1136/bmjopen-2017-018580 29615443 PMC5892750

[19] Mackenzie D Pfitzer A Maly C: Data from: Postpartum family planning integration with maternal, newborn, and child health services: a cross-sectional analysis of client flow patterns in India and Kenya. *Dryad Digital Repository.* 2018 10.5061/dryad.11313 PMC5892750 29615443

[20] Pfitzer A Kabue M: PPFP Integration in India and Kenya.2019 10.17605/OSF.IO/5TFA7

[21] Birdthistle IJ Mayhew SH Kikuvi J: Integration of HIV and maternal healthcare in a high HIV-prevalence setting: analysis of client flow data over time in Swaziland. *BMJ Open.* 2014;4(3):e003715. 10.1136/bmjopen-2013-003715 24607560 PMC3948459

[22] Coker R Balen J Mounier-Jack S: A conceptual and analytical approach to comparative analysis of country case studies: HIV and TB control programmes and health systems integration. *Health Policy Plan.* 2010;25 Suppl 1:i21–31. 10.1093/heapol/czq054 20966105

[23] Topp SM Chipukuma JM Chiko MM: Integrating HIV treatment with primary care outpatient services: opportunities and challenges from a scaled-up model in Zambia. *Health Policy Plan.* 2013;28(4):347–57. 10.1093/heapol/czs065 22791556 PMC3697202

[24] Gounder CR Chaisson RE: A diagonal approach to building primary healthcare systems in resource-limited settings: women-centred integration of HIV/AIDS, tuberculosis, malaria, MCH and NCD initiatives. *Trop Med Int Health.* 2012;17(12):1426–1431. 10.1111/j.1365-3156.2012.03100.x 23113824

[25] Pfitzer A Mackenzie D Blanchard H: A facility birth can be the time to start family planning: postpartum intrauterine device experiences from six countries. *Int J Gynaecol Obstet.* 2015;130 Suppl 2:S54–61. 10.1016/j.ijgo.2015.03.008 26115859

[26] Lawn S Lloyd A King A: Integration of primary health services: being put together does not mean they will work together. *BMC Res Notes.* 2014;7:66. 10.1186/1756-0500-7-66 24479605 PMC3915222

[27] Achyut P Mishra A Montana L: Integration of family planning with maternal health services: an opportunity to increase postpartum modern contraceptive use in urban Uttar Pradesh, India. *J Fam Plann Reprod Health Care.* 2016;42(2):107–15. 10.1136/jfprhc-2015-101271 26622056 PMC4853573

[28] Yadav D Dhillon P: Assessing the impact of family planning advice on unmet need and contraceptive use among currently married women in Uttar Pradesh, India. *PLoS One.* 2015;10(3):e0118584. 10.1371/journal.pone.0118584 25738707 PMC4349805

[29] Wilcher R Hoke T Adamchak SE: Integration of family planning into HIV services: a synthesis of recent evidence. *AIDS.* 2013;27 Suppl 1:S65–75. 10.1097/QAD.0000000000000051 24088686

[30] Mayhew SH Ploubidis GB Sloggett A: Innovation in Evaluating the Impact of Integrated Service-Delivery: The Integra Indexes of HIV and Reproductive Health Integration. *PLoS One.* 2016;11(1):e0146694. 10.1371/journal.pone.0146694 26800517 PMC4723242

[31] Kumar S Sethi R Balasubramaniam S: Women's experience with postpartum intrauterine contraceptive device use in India. *Reprod Health.* 2014;11:32. 10.1186/1742-4755-11-32 24755312 PMC4062773

[32] BellowsBWConlonCMHiggsES: A taxonomy and results from a comprehensive review of 28 maternal health voucher programmes. *J Health Popul Nutr.* 2013;31(4 Suppl 2):106–28. 24992806

[33] Sweeney S Obure CD Terris-Prestholt F: The impact of HIV/SRH service integration on workload: analysis from the Integra Initiative in two African settings. *Hum Resour Health.* 2014;12:42. 10.1186/1478-4491-12-42 25103923 PMC4130428

[34] Church K Wringe A Fakudze P: The relationship between service integration and client satisfaction: a mixed methods case study within HIV services in a high prevalence setting in Africa. *AIDS Patient Care STDS.* 2012;26(11):662–73. 10.1089/apc.2012.0191 23078548

[35] Calhoun LM Speizer IS Rimal R: Provider imposed restrictions to clients’ access to family planning in urban Uttar Pradesh, India: a mixed methods study. *BMC Health Serv Res.* 2013;13(1):532. 10.1186/1472-6963-13-532 24365015 PMC3879325

[36] Ryman TK Wallace A Mihigo R: Community and health worker perceptions and preferences regarding integration of other health services with routine vaccinations: four case studies. *J Infect Dis.* 2012;205 Suppl 1:S49–55. 10.1093/infdis/jir796 22315386

[37] Cairns KL Perry RT Ryman TK: Should outbreak response immunization be recommended for measles outbreaks in middle- and low-income countries? An update. *J Infect Dis.* 2011;204 Suppl 1:S35–46. 10.1093/infdis/jir072 21666184

