# Supplementary material for: Characteristics of successful integrated family planning and maternal and child health services: Findings from a mixed-method, descriptive evaluation
Source: F1000Res. 2020 Jan 13;8:229. Originally published 2019 Feb 28. [Version 2] doi: 10.12688/f1000research.17208.2 (PMC6993833; doi:10.12688/f1000research.17208.2)
Supplement: Peer review history

# Peer review: Reviewer response for version 2

**DOI:** 10.5256/f1000research.24085.r58544

**Published:** 2020-02-07

**Authors:** Marina A.S. Daniele

**Affiliations:** 1 King's College London, London, UK

No further comments to make.

I confirm that I have read this submission and believe that I have an appropriate level of expertise to confirm that it is of an acceptable scientific standard.

# Peer review: Reviewer response for version 2

**DOI:** 10.5256/f1000research.24085.r58545

**Published:** 2020-01-28

**Authors:** Ilene S Speizer

**Affiliations:** 1 Department of Maternal and Child Health, Gillings School of Global Public Health, "University of North Carolina at Chapel Hill, Chapel Hill, NC, USA

I confirm that I have read this submission and believe that I have an appropriate level of expertise to confirm that it is of an acceptable scientific standard.

# Peer review: Reviewer response for version 1

**DOI:** 10.5256/f1000research.18817.r51874

**Published:** 2019-08-27

**Authors:** Ilene S Speizer

**Affiliations:** 1 Department of Maternal and Child Health, Gillings School of Global Public Health, "University of North Carolina at Chapel Hill, Chapel Hill, NC, USA

This paper examines integration levels of MNCH and FP services in hospitals in two states in India and a hospital and health centers in two counties in Kenya. The strength of the paper is the use of multiple data sources (qualitative and quantitative) to inform the analysis on an important topic. A limitation of the paper is the small sample of facilities in each country which means it is not possible to determine if some facilities (e.g., hospitals or health centers) are more amenable to integration than others. Related to this is that the selection of facilities suggests a preference for higher performing (on integration) facilities and thus, even though not all the included facilities were highly integrated, the results cannot be generalized across other similar types of facilities.  An additional limitation includes that it is not possible to distinguish whether the findings are indicative of higher/lower integration and/or higher/lower quality of services. Quality discussions are notably absent from the paper and might be worth including since integration is a component of higher quality services. Below I highlight a few other comments:

  

1. The authors mention the India government initiative to have dedicated PPFP counselors in health facilities. The authors fail to note if the included India facilities have a dedicated counselor or not and if this is correlated with the level of integration observed in this study. 
2. More detail is needed on the measurement of integration. The description of “as done in other studies” is vague and not informative enough for this paper.
3. Table 1 – the median age of participants is provided – is this for clients or for all study participants including providers and key informant interviews? I expect this is just for clients and thus this should be clarified.
4. Table 2 – you talk about “dark gray” but in the formatted version of the table, it is not clear what is “dark gray.” Also, do you need a “facility overall” column that is inferred in the text?
5. For the quotes, it would be useful to know if the respondent is in a high, medium or low integrated facility. This is included with some but not all the quotes.
6. The text starts with “All MNCH clients” when it is discussing Table 3, however, Table 3 only includes ANC clients. Make sure this is clear in the text (and possibly explain why only the ANC clients are shown). 
7. The consistent and affordable supply of FP and the provider bias sections are particularly relevant to the quality of care issue mentioned above.  Quality could be raised in the introduction to help tie integration to quality and the issues in this section. 
8. The explanation of the inconsistent results in India relate to client volume (which makes sense), however, this is the first time that client volume is mentioned in the paper. Given that all facilities in India are hospitals, I wondered how client volume plays into the results. Can client volume be discussed earlier. Also, are some of these hospitals private and others public and does that affect integration (and quality)? For Kenya, it explicitly says that public facilities were included but this detail was not found for India. 

I confirm that I have read this submission and believe that I have an appropriate level of expertise to confirm that it is of an acceptable scientific standard, however I have significant reservations, as outlined above.

# Response

**Published:** 2019-12-20

**Authors:** Anne Pfitzer

**Affiliations:** Jhpiego, USA

Thank you for including an assessment of strengths and limitations in your review. We acknowledge the limitation of a small number of facilities in each country. Also, we selected facilities that received programmatic support, thus we can be very clear that finding on integration are not generalizable. In fact, we believe our findings suggest that programs to encourage integration take hold unevenly across facilities, and we have sought to better understand how to encourage greater effective integration of services.

 We respectfully dispute your assertion that integration is a component of higher quality services. We looked at traditional quality measures across levels of integration (and in fact there may be a tradeoff in quality with greater integration if not careful), but considered integration a distinct aspect of service delivery. That being said, we added a sentence to the introduction highlighting parallels between integration and quality.

 We would have liked to respond more specifically to the suggestion about clarifying whether all study facilities had a counsellor. However, we did not collect this information. We can report that we had transcripts of qualitative interviews with counsellors in 4 out of the 6 Indian hospitals.

 The comment about detail on measurement of integration is consistent with other reviewers. Our response is also the same.

 We decided that it is not appropriate to include a note about the median age of clients in Table 1 and removed it.

 The suggestion about detailing the respondents facilities’ level of integration for the qualitative quotes echoes the 2nd reviewer. We have added this.

 Thank you for the keen observations about inconsistencies. Specifically,

1. About the legend of Table 2 – the text was not amended when we changed the formatting of the table. This has been corrected.
2. Discrepancies between the text and Table 3. All MNCH clients were asked that question, but we only report on ANC clients because of small numbers of PNC and child health clients (indicated in methods section). We have revised.
3. We added a statement about client volume in the sample description of the methods section.
4. The lack of precision about public vs private settings. We have added a clarification that all facilities in this study were public in the sampling section of the methods.

# Peer review: Reviewer response for version 1

**DOI:** 10.5256/f1000research.18817.r51879

**Published:** 2019-08-27

**Authors:** Marina A.S. Daniele

**Affiliations:** 1 King's College London, London, UK

This study is a timely and important effort to expand on the evidence base for successful PPFP integration with maternal and newborn health services. The attempt to evaluate programmatic experiences in a systematic way is commendable.

 The researchers have a good knowledge of existing evidence and gaps and display this clearly in the introduction and discussion.

 The methods section begins with an appropriate context setting section, describing integration programmes in the study sites. As done here, it is often useful for triangulation purposes to combine qualitative and quantitative methods. The client flow method used appears to be robust and cost-effective. The semi-structured interviews also appear to be sound in design and quality. As pointed out by the authors themselves in the discussion, observation of care would have provided important insights.

 However, I have doubts concerning the quality of the data generated from the provider and client surveys. The numbers are fairly small, and there is a notable risk of courtesy bias. In this regard, I am particularly curious about the extent to which providers, especially, were aware that the researchers were assessing family planning integration, and whether they felt they could be completely honest about the extent to which they did or did not provide PPFP services. It would be useful for the authors to comment on these issues in the discussion. The area I am most uncertain about is the ability of the data on privacy, waiting time and interactions with staff to support the kind of analysis presented. The numbers appear to be very small, so I am not sure that they support quantitative comparisons between facilities. Qualitative interviews with clients might have been a better way to explore their experience of care.

 In addition, it is difficult to express a full opinion about the soundness of all the analysis done because of a somewhat confusing presentation of methods and results. I had to read the methods section several times in order to understand what was done, with what purpose, and in what order. Sentences describing the purpose of each phase, and step, should precede those describing which type of data was considered in turn.

 The development of an integration score for facilities is an essential part of the analysis, but different (more or less collated or aggregated by service area) classifications of integration levels are described in the methods, presented in Table 2, and referred to in the results section. I also could not find a clear explanation about why, out of 15 facilities in the sample, integration scores are only presented for 13.

 While containing interesting findings, the results section could be reorganised to be more clear, perhaps with more correspondence with the analysis phases as described in the methods, e.g. into three subject areas rather than two, the third being client experience. As it is, the second section of the results (Factors influencing success) appears quite long and difficult to navigate. However, I am not certain that the parts on client experience should be retained, given the reservations about data quality that I expressed above.

 Although, it could benefit from some streamlining and re-organisation, the Discussion reflects the strengths and weaknesses of the study and discusses the findings in the light of relevant contextual factors which aid the interpretation. The piece could be improved by giving more prominence to the paragraphs discussing what the implications for policymakers and future researchers are.

I confirm that I have read this submission and believe that I have an appropriate level of expertise to confirm that it is of an acceptable scientific standard, however I have significant reservations, as outlined above.

# Response

**Published:** 2019-12-20

**Authors:** Anne Pfitzer

**Affiliations:** Jhpiego, USA

Thank you for your acknowledgement of the value of reporting on program evaluations. We also appreciate the reviewer taking time to provide a summary of the paper.

 We added a sentence to the Discussion section, noting potential for courtesy bias in provider interviews.

 The consent form did describe the purpose of the study: “We are trying to understand how family planning is included with other health services at this facility particularly maternal, newborn child health and nutrition services.“ and …”ask you a series of questions  related to maternal, newborn, child health and nutrition services offered at this facility, the inclusion of family planning in these services and what impact the inclusion of family planning has had ….a) on these services and in general at this facility (providers, incharges) or b) …on you as a client (clients)”. However, we also assured them that we were not recording their names or identifying facilities so there was no way to link their statements to them and we would not report results by facility name. The Study Information Sheet given to in charges and posted in facilities stated: “The aim of this study is to strengthen the body of programmatic learning around the integration of PPFP services into maternal, newborn, child health and nutrition services. It is a descriptive evaluation that will investigate and compare details on services for pregnant women and those who have had a child in the last 2 years and how they integrate aspects of maternal and infant young child nutrition and/or family planning in selected facilities and community programs in Bondo and Embu sub-counties in Kenya and Jharkhand and Bihar states in India. The objectives of the study are to describe implementation models, to understand barriers and facilitating factors related to the implementation and scale up of each model, and to describe client perceptions of each model.”

 The previous reviewer also commented on scoring for integration and waiting time and privacy. However, the methods described that analysis was in phases, with the first phase on levels of integration, phase 2 relating qualitative results to whether facilities are well integrated or not, and phase 3 the effect of these models of integration on clients experience of care. We have reorganized the results to include a clearer section on experience of care.

 We acknowledge the small number of respondents, which adds a degree of uncertainty around the responses regarding aspects of quality of care such as privacy and waiting times. The reviewer is correct that qualitative interviews with client may have provided richer understanding of the quantitative data. However, we have little reason to doubt clients who expressed concerns about the services they received. As the reviewer pointed out, courtesy bias would suggest under-reporting of unsatisfactory or nuisances during a health visit.

 The concerns about the presentation of methods are similar to the previous reviewer’s. We have sought to address this by adding a text box to further describe the methodology, including the specific questions which were used to assess levels of integration.

 As to why only 13 facilities have integration scores, the reviewer likely missed a sentence under the Data Management and analysis section (paragraph with heading “FP integration and qualitative data analysis” in bold) which states: “We excluded two facilities from Kenya where we did not collect client flow data because they had fewer than five client respondents per service area.”  In addition, Table 2 notations point to two facilities labeled KO03 and KO06 who are excluded and the reasons why are stated.

 We agree that re-organizing the Results section to reflect the phases of our analysis makes sense. Thank you for the suggestion. We appreciate the concerns about the small numbers of clients for the client experience phase of the analysis. However, we choose to retain the data on client experiences since our study did not include qualitative interviews with clients to obtain their perspectives, and those perspectives are important.

 We added a statement to introduce the discussion which seeks to frame the implications for policymakers.

# Peer review: Reviewer response for version 1

**DOI:** 10.5256/f1000research.18817.r46707

**Published:** 2019-06-10

**Authors:** Priya Nanda

**Affiliations:** 1 Equity and Social Change, Research and Evaluation, Bill & Melinda Gates Foundation, New Delhi, Delhi, India

This is a very interesting study that draws its interpretations from a very small sample in Kenya and India. They compare the state on integration and quality of services across different size facilities in each study sites. The data is biased as it draws on high client load in well integrated facilities than poorly integrated facilities. Providers who were available were interviewed but there is no information whether these are the providers who were also trained in PPFP. 

 It is not clear why the research team did not use facility client flow data over the last 3-4 weeks as a robust way to understand flows rather than through observation during the says of study visit.  

 I like the approach of hierarchy of evidence and when there is disagreement between expert analyse and client flow giving preference to client flow data. Can the researchers explain when and in what instances did these disagreements happen? What might be other factors that the experts were looking at—this might help in understanding what are other metrics for well integration that the study could have looked at?

 The definition of integration in this study may be limited. Would the authors like to reflect on how else they may have defined and measured it? For example they look at whether the client at ANC or other departments is getting FP counselling. What about capacity of counsellor to counsel on PPFP—is that a given that its is good in well integrated facilities?  Is there more informed choice and method availability in well integrated facilities?

 It would be helpful if the authors provide briefs on how scoring was done for integration. I see that they looked at wait times and privacy but its not clear to me whether these were attributes of well integrated or outcomes at well integrated facilities?

 When a facility is badly integrated, does it get lower client load—is there a way to say that from the experience of well and medium integrated facilities? Is it possible to get high client load at facilities that are more crowded but yet are badly integrated where client flow pathways are more complicated?

 In this study, its difficult to discern what went into the analysis as a definition of integration and what from the analysis is coming out as a determinant of integration? 

 The results are interesting yet the methodology is unclear and seems a bit bias-what the authors report as determinants are also definitions of what is coded as well integrated?

 Bihar in fact shows contrary results where privacy and waiting times are worse in well integrated facilities. These results needs their own discussions—its possible that well integrated services take longer time but privacy cannot be lower in well integrated facilities? Or it can if integration is only about offering services at different points of contact.

 In order to contextualize the information from respondents, it would be useful if the qualitative information is cited from coming from a well or poorly integrated facilities. For example, in facilities where providers are talking about poor counselling or poor training, are those also facilities that were coded as poorly integrated? 

 The article brings out issues of provider bias in the section on context. This is a key issue that needs to emphasised, as they clearly suggest that integrated services do not necessarily address provider bias or rights adequately. 

 The authors discuss the limitations of the study well which is one reason I am not bringing it up in my review. However their discussion draws heavily from other studies, as this study itself presents inconclusive and mixed evidence. I think the study would be stronger if we started the analysis with  fixed parameters on which questions were asked and then triangulated the qualitative data to match whether the fixed parameters for well integrated match with qualitative analysis of attributes of good quality services (which may or may not have high correlation with integration).

I confirm that I have read this submission and believe that I have an appropriate level of expertise to confirm that it is of an acceptable scientific standard, however I have significant reservations, as outlined above.

# Response

**Published:** 2020-01-08

**Authors:** Anne Pfitzer

**Affiliations:** Jhpiego, USA

Thank you for your thoughtful review.

In response to the issue of bias because higher client load in well-integrated facilities than poorly integrated ones: We should clarify that we had more clients surveyed in Indian well-integrated facilities, but this is not necessarily indicative of client loads. However, this is an interesting observation nevertheless. The purposive selection of facilities clearly had limitations, but client load was not a criterion for the study (although high volume of deliveries was a criteria for PPFP program support). While this may introduce bias, we were surprised about this finding as we would think that facilities with higher loads would have more difficulty with integration. We did find we had to make a correction and clarify that the issue of client respondents was specific to India, not Kenya.

Regarding information as to whether provider interviewed were trained in PPFP: we asked providers whether they had confidence in their abilities, whether they had the skills to do it, and whether they received sufficient support, but not if they were trained. We chose to report on more direct indications of provider performance and did not examine relationship between self-reported training experience, confidence, skills, support and level of integration.

For the client flow methods, we chose one week as we felt it would address any fluctuations due to weekly occurrences, such as market days. While more client flow data might have been more robust, this was the amount of time feasible within project resources.

On the approach of hierarchy of evidence and analysis of levels of integration: additional text and text box have been added to provide further detail and transparency in classification of facilities. Disagreements occurred in cases where provider and client reports were inconsistent with each other and small numbers of respondents presented challenges for comparison; this was addressed by establishment of decision rules regarding inclusion criteria and hierarchy of data sources.

The reviewer makes a valid comment about the limitations of looking strictly at integration of services without also addressing the quality of services, which would have required an observation. We do not have such information, only that all facilities received roughly equivalent program support within the programs described in the paper.

On the issue of wait times and privacy, we should also note that wait times and privacy were not part of the determination of integration, but we sought instead to understand whether the level of integration had an effect on clients’ reporting of these attributes of services. But in reference to a later comment, we have added a bit more interpretation of the findings regarding privacy issues in the discussion section. Indeed our findings suggest that integration and quality are not entirely correlated, but this may be partly because a comparison of the effects of integration on quality is an imperfect one as we didn’t ask clients a counterfactual of whether they would have preferred longer wait times for example in exchange for additional services provided during the visit.

The reviewer’s questions about levels of integration and client load are interesting questions. However, we do not feel our dataset can answer them.

We appreciate the need to contextualize the qualitative responses, so we went back to the transcripts, in order to re-identify all the quotes and classified them. We have thus clarified whether they were from well-integrated or poorly integrated facilities. In the process, we had to make additional corrections when quotes were mislabeled. In one or two cases, we made cuts because some quotes may have been misinterpreted when segmented out of context.

The review also makes a good point about provider bias related to context. We have added a sentence about bias as a separate issue to integration.

We found the feedback about the questions asked very helpful and have added a text box outlining the questions used in the analysis of integration. As noted, we clearly outline the limitations of this study and highlight complexities to be considered in future evaluations of family planning integration in other health services.
